# Seasonal cycles of the TBE and Lyme borreliosis vector *Ixodes ricinus* modelled with time-lagged and interval-averaged predictors

**DOI:** 10.1007/s10493-017-0197-8

**Published:** 2017-11-27

**Authors:** Katharina Brugger, Melanie Walter, Lidia Chitimia-Dobler, Gerhard Dobler, Franz Rubel

**Affiliations:** 1 Institute for Veterinary Public Health, University of Veterinary Medicine Vienna, Veterinärplatz 1, 1210 Vienna, Austria; 2 Bundeswehr Institute of Microbiology, Neuherbergstraße 11, 80937 Munich, Germany; 3 German Center of Infection Research (DZIF) Partner Site Munich, Munich, Germany; 4 Parasitology Unit, University of Hohenheim, Emil-Wolff-Straße 34, 70593 Stuttgart, Germany

**Keywords:** Castor bean tick, Cross-correlation map, Climate, Hunting statistics, Tick-borne diseases

## Abstract

Ticks of the species *Ixodes ricinus* (L.) are the major vectors for tick-borne diseases in Europe. The aim of this study was to quantify the influence of environmental variables on the seasonal cycle of questing *I. ricinus*. Therefore, an 8-year time series of nymphal *I. ricinus* flagged at monthly intervals in Haselmühl (Germany) was compiled. For the first time, cross correlation maps were applied to identify optimal associations between observed nymphal *I. ricinus* densities and time-lagged as well as temporal averaged explanatory variables. To prove the explanatory power of these associations, two Poisson regression models were generated. The first model simulates the ticks of the entire time series flagged per 100 m$^2$, the second model the mean seasonal cycle. Explanatory variables comprise the temperature of the flagging month, the relative humidity averaged from the flagging month and 1 month prior to flagging, the temperature averaged over 4–6 months prior to the flagging event and the hunting statistics of the European hare from the preceding year. The first model explains 65% of the monthly tick variance and results in a root mean square error (RMSE) of 17 ticks per 100 m$^2$. The second model explains 96% of the tick variance. Again, the accuracy is expressed by the RMSE, which is 5 ticks per 100 m$^2$. As a major result, this study demonstrates that tick densities are higher correlated with time-lagged and temporal averaged variables than with contemporaneous explanatory variables, resulting in a better model performance.

## Introduction

It is undeniable that ticks and their ability to transmit medically relevant pathogens play an important role for public health. The most important examples are the tick-borne encephalitis (TBE) virus or *Borrelia burgdorferi* sensu lato, the complex of bacteria causing Lyme borreliosis (LB). Reviews on TBE and LB concerning the study region Central Europe (Germany) have recently been published on the epidemiology and distribution of TBE (Dobler et al. 2012), the progress in TBE research (Kunze and The ISW-TBE 2016) and LB in general (Stanek et al. 2012). To contribute to an adequate TBE and LB risk assessment, which should incorporate the phenology of the vectors involved (Norman et al. 2016), an enhanced method to determine variables explaining the seasonal cycles of ticks is introduced. The focus is on the main vector *Ixodes (I.) ricinus*, which is widely distributed in Germany (Rubel et al. 2014).

Brugger et al. (2016) compiled a dataset of 69 German sites from which monthly *I. ricinus* time series were collected. However, most of these time series are only 1–2 years long and so unsuitable to depict inter-annual tick fluctuations. The longest time series in Haselmühl (Germany) lasting eight consecutive years without data gaps, was used in this study. As all the other tick time series compiled by Brugger et al. (2016), the number of ticks (abundance) was related to the same flagging area of 100 m$^2$. Thus, ticks were given as densities in units 1/100 m$^2$. To quantify the seasonal and inter-seasonal activity of ticks usually climatic variables as temperature or precipitation were used (e.g. Cat et al. 2017; Schulz et al. 2014). Contrary to the monthly tick time series, such variables are observed on a regular base (previously several times per day, meanwhile every minute) under defined standards (World Meteorological Organization 2008). Some time-series last over more than two centuries, e.g. in Germany the stations Berlin or Hohenpeißenberg.

So far the majority of studies used climatic variables on the sampling day to explain influences on the seasonal tick density (Daniel et al. 2015; Berger et al. 2014). In some cases, a possible time-lagged association of climate variables up to 8 days prior to the sampling event was considered (Barandika et al. 2006; Kiewra et al. 2014; Li et al. 2012). However, in all studies, the influence of these short time lags was determined not to be significant. Only Kazimírová et al. (2016) reported a negative correlation between nymphal density and the mean saturation deficit of the preceding 2 months. More frequently temporal accumulated or averaged climate variables have been used to statistically explain tick seasonality (Perret et al. 2000; Alonso-Carné et al. 2016; Osipova et al. 2017). Alternatively, the effect of the winter conditions on the subsequent tick season was conceived by defining temperature or relative humidity thresholds (Dautel et al. 2008; Vollack et al. 2017).

Here, an enhanced method of the classical correlation analysis, frequently used to determine the influence of environmental variables, is applied. With these cross correlation maps (CCMs), optimal associations between ticks sampled during a specific flagging event and time-lagged as well as interval-averaged environmental variables were identified. Initially developed for mosquitoes of the genus *Aedes* spp. (Curriero et al. 2005; Shone et al. 2006), this method has also been applied for *Culex* spp. (Walsh et al. 2008; Chuang et al. 2012; Lebl et al. 2013; Lockaby et al. 2016; Groen et al. 2017), biting midges such as *Culicoides* spp. (Brugger and Rubel 2013; Diarra et al. 2015), and stable flies such as *Stomoxys calcitrans* (Taylor et al. 2007; Taylor and Berkebile 2011). Recently, CCMs have also been used to explain the seasonal dynamics of dengue (Stoddard et al. 2014) and to detect abortive diseases in cattle (Bronner et al. 2015).

Here, this method is applied for the first time to ticks (nymphal *I. ricinus*). As the life cycle of *I. ricinus* comprises four life stages (egg, larva, nymph, and adult) and lasts up to several years (Gray et al. 2016), time-lagged influences should be considered for a comprehensive analysis of tick dynamics. Within this usually 3- to 4-year period, key processes determining the tick dynamics such as development (rate and duration, emergence), diapause (survival, inactivity) and questing (activity, ability to find host, survival) are affected by environmental variables (Ostfeld and Brunner 2015). This study aims to quantify the time-lagged and interval-averaged influence of environmental variables on tick density and, secondly, to simulate the seasonal and inter-annual density fluctuations for a disease risk assessment.

## Materials and methods

### Study site and tick flagging

**Fig. 1:**
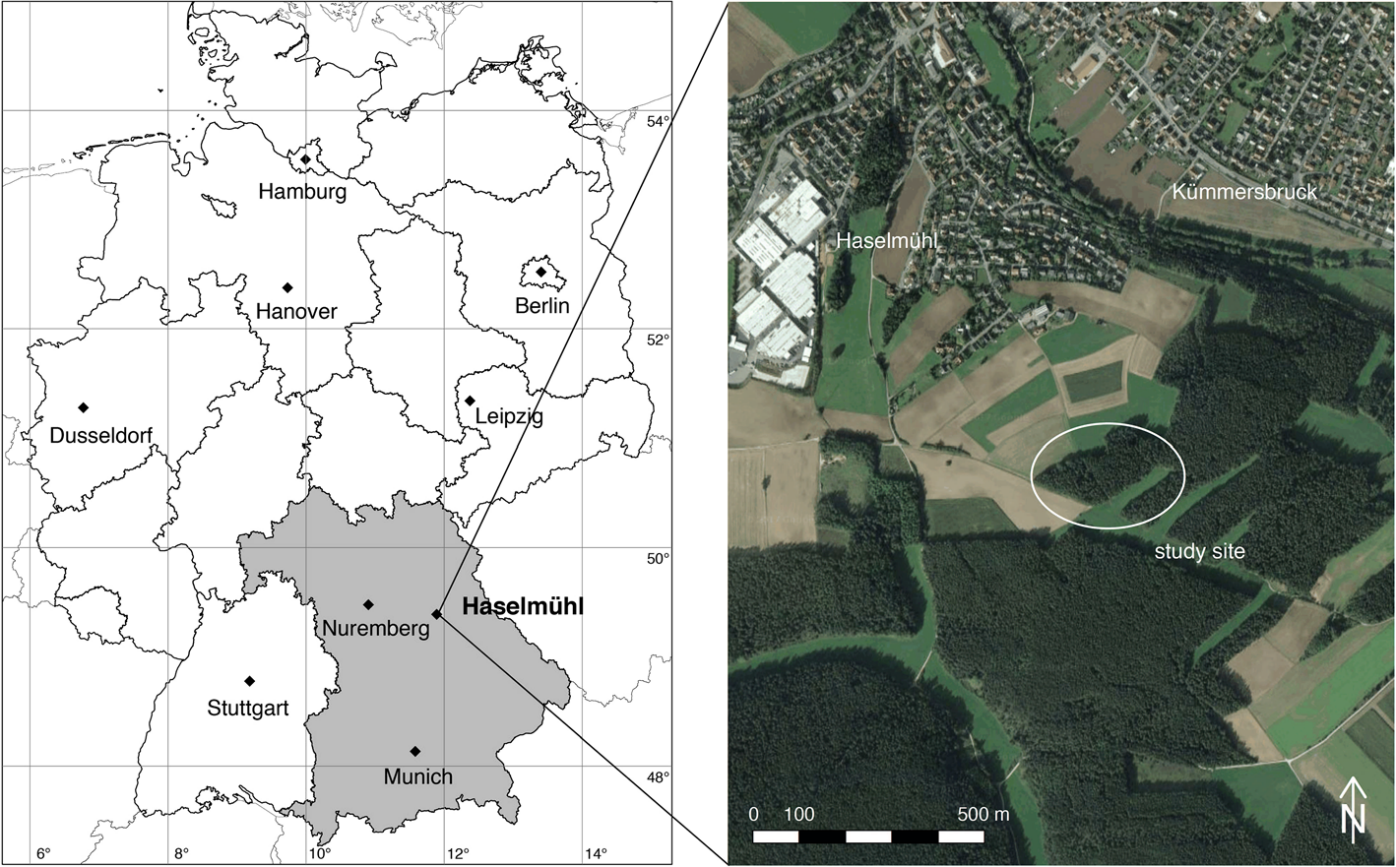
The flagging site Haselmühl is located in the southeast of Germany in the Bavarian district Amberg-Sulzbach (left) and is a rural area characterised by arable land, forests, and scattered villages (right)

The site Haselmühl is one of the most intensively studied natural foci of tick-borne encephalitis (TBE) in Germany (Weidmann et al. 2013). This rural area in the administrative district of Amberg-Sulzbach is around 60 km east of Nuremberg, the second largest city in the German federal state of Bavaria (Fig. 1). The site is located 430 m above sea level at geographic longitude 11.8819$^\circ$E and latitude 49.4083$^\circ$N. The natural focus is characterised by arable fields surrounded by mixed forests with a predominance of pines (*Pinus sylvestris*) and dense undergrowth.

Between March 2009 and October 2016, questing *I. ricinus* (larvae, nymph, adults) were collected at monthly intervals using the flagging method. Ticks were always flagged 2 h before dawn at the last weekend of the month or, if weather conditions were unsuitable, on the following weekend. A $50\times 100$ cm flag of cotton cloth was flagged over the low vegetation along a standardised 800 m trail. The flagged densities were, therefore, the number of questing ticks per 400 m$^2$. The flagging was usually done by two people, each one sampling for 45 min, or by one person sampling for 90 min. The collected ticks were identified under a Zeiss Stemi DV4 microscope using the identification key according to Hillyard (1996).

### Explanatory variables

In this study, climate  variables were obtained from the nearest weather station Regensburg-Oberhub (WMO No. 107760) of the German Weather Service (2017). The station is located at the geographic coordinates 12.1021$^\circ$E/49.0424$^\circ$N in 365 m above sea level and around 16 km away from the flagging site Haselmühl. The latter is characterised by a warm temperate climate with rain throughout the year, classified as Cfb climate according to Köppen-Geiger (Kottek et al. 2006). This climate is optimal for deciduous and mixed forests (Rubel et al. 2017), which are known for their high *I. ricinus* density (Boehnke et al. 2015; Brugger et al. 2016). For the period 2008–2016, monthly time series of temperature and relative humidity, were aggregated out of daily measurements. Additionally, 30-year monthly averages (1985–2014) of each variable were calculated.

Another environmental variable affecting tick densities is the availability of suitable hosts. Nymphal ticks mainly feed on small to medium-sized animals, such as rodents, hares or hedgehogs. This is contrary to the behaviour of adults, which prefer to feed on large-sized animals like roe deer (Gray et al. 2016). Although roe deer density is a good site-specific predictor for TBE (Rizzoli et al. 2009), it does not significantly change over time and was, therefore, not suitable for use in the model. However, as no data on the densities and inter-annual changes of rodents were available for Haselmühl, hunting statistics of another host, the European hare (*Lepus europaeus*), were used. These lagomorphs are known to be both competent reservoir and blood hosts for all tick life-stages, but especially for larvae, in environments lacking rodents (Tälleklint and Jaenson 1993). The Bavarian Ministry of Food, Agriculture and Forestry provides annual hunting statistics for administrative districts via the Wildtierportal Bayern (http://www.wildtierportal.bayern.de). In Bavaria, the official hunting season for hares runs from the 15th October to 31st December.

Tick densities may also vary between years or seasons of the year due to unknown or non-measurable variables. Examples include the availability of suitable hosts for each life stage (Gray 1998), higher mortality rate in the winter months (Gray 1981) or rodent cycles (Mihalca and Sándor 2013). Therefore, the season was included as a categorical variable (classes I–IV). Note that the meteorological season with groups of 3 months based on the annual temperature cycle was used, e.g. meteorological spring includes March, April, and May.

### Statistical analysis

**Fig. 2:**
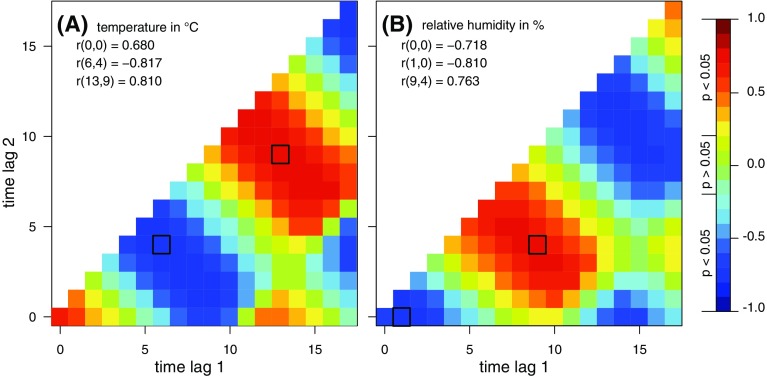
Cross correlation maps (CCMs) of the monthly time series of nymphal ticks versus both (**a**) temperature in $^\circ$C and (**b**) relative humidity in %. The correlation coefficient for the month of the flagging event r(0, 0) as well as the minimum and maximum time-lagged correlation coefficients r(lag$_1$, lag$_2$) are given. The tick density is maximal negatively correlated with the temperature averaged from 6 to 4 months and the relative humidity averaged from 1 to 0 months prior the flagging event. Significance levels depending on the constant sample size of n = 91 and the floating correlation coefficient r indicates that all values $\vert r \vert\,\ge\,0.206$ are significant ($p\,<\,0.05$). Period: 2009–2016

The method of cross correlation maps (CCMs) was applied to determine the environmental variables and their highest correlation with tick densities on defined time lags. As an improvement on the ordinary cross correlation, not only the month-to-month correlation between the tick densities and an explanatory variable were obtained, but also time-lagged and interval-averaged correlations by considering a second time lag. The CCM in Fig. 2 (left) shows a maximum correlation between the tick density and, for example, the temperature averaged from time lag 1 (e.g. 13 months) to time lag 2 (e.g. 9 months) prior the flagging event. The CCM method was previously explained in detail by Brugger and Rubel (2013) and the R source code is provided on the website http://epidemic-modeling.vetmeduni.ac.at/. Based on the findings, a data set comprising environmental variables (e.g. temperature or hunting statistics of hares) was compiled. Finally, two Poisson regression models were developed to simulate the inter-annual tick density of the complete time series (model I) and the mean seasonal cycle (model II). To account for the overdispersion of the data, the standard errors were corrected using a quasi GLM model where the variance is specified by the mean and the dispersion parameter (Zuur et al. 2009). The Akaike information criterion (AIC; Akaike 1974) was used as variable selection criterion. Non-significant variables were removed in a stepwise procedure. The coefficient of determination for generalised linear models $R^2$ introduced by Zhang (2017) and the root mean square error (RMSE) were selected as goodness-of-fit measures.

All analyses were conducted with the open-source statistical computing environment R (R Development Core Team 2017). The package rsq (Zhang 2016) was used for calculating the coefficient of determination $R^2$.

## Results

A total of 473 larvae, 8636 nymphs, and 3819 adult *I. ricinus* ticks (1897 female, 1922 male) were flagged over 8 years of consecutive sampling (2009–2016). However, as nymphs are the most important stage for pathogen transmission to humans (Gray 1998), only this life stage was considered here. As mentioned above, ticks were collected by flagging an area of 400 m$^2$. To allow for comparison with the more commonly used area of 100 m$^2$, tick numbers were divided by 4.

The cross-correlation maps revealed that temperature (T) and relative humidity (rH) were highly correlated with nymphal tick densities (Fig. 2). In accordance with the four life stages of *I. ricinus*, a broader time frame of the 18 months preceding the flagging event was considered. For each explanatory variable, the highest positive and negative time-lagged and interval-averaged correlations as well as the month-to-month correlations T(0, 0) and rH(0, 0) were determined. For example, the highest negative correlation of r(6, 4) = − 0.817 was found for ticks versus temperature averaged over 4–6 months prior to the flagging event, while the highest positive correlation of r(13, 9) = 0.810 was found for temperatures averaged from 13 to 9 months prior to flagging.

**Fig. 3:**
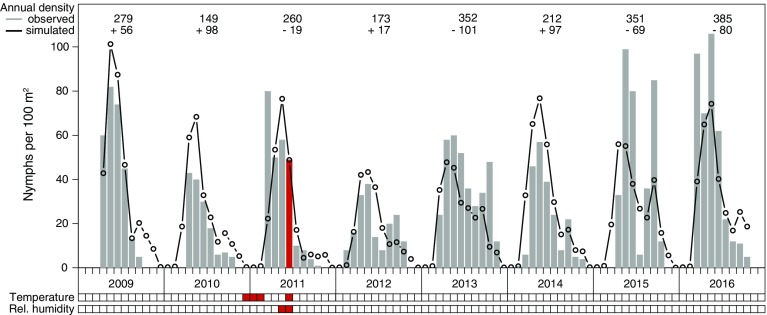
Monthly nymphal tick density in Haselmühl (Germany) between 2009 and 2016 (unit: nymphs per 100 m$^2$). Observations are shown as grey bars and simulations as lines. To illustrate the climatic variables determining the density in May 2011, the mean temperature between November 2010 and January 2011 as well as the mean relative humidity from April to May 2011 are highlighted in red

A set of time series including the monthly temperature and humidity values T(0, 0) and rH(0, 0), as well as the time-lagged values identified with the highest positive correlation, T(13, 9) and rH(9, 4), and negative correlation, T(6, 4) and rH(1, 0), was compiled. The set was then incorporated into the regression analysis together with the annual values of the hare hunting statistics and the seasons I–IV as categorical value. Non-significant variables were removed stepwise to select the model with the lowest Akaike information criterion (AIC). The best fitted model included the month-to-month temperature T(0, 0), the mean relative humidity of the actual month and 1 month previously rH(1, 0), the averaged temperature over 4–6 months prior to the flagging event T(6, 4), the hare hunting statistics (hare) shifted by 1 year, and the season as a categorical variable (classes I–IV) as summarized in Table 1. As depicted in Fig. 3, the model simulated the seasonal cycle as well as the inter-annual fluctuations reasonably well. Little to no activity was observed in the winter months, while the activity peaked in late spring, mainly in May. In some years, a secondary, but not so marked, peak was also documented in early autumn, mainly in September. The coefficient of determination $R^2$ indicated that a total of 64.8% of the variation in the observed nymphal density was explained by the model. Compared to the null model with exclusively climatic variables (not shown), the $R^2$ is 27% higher. The goodness-of-fit was evaluated by a RMSE of 17 ticks per month.

**Table 1:** Summary of the Poisson regression models for inter-annual tick density of the complete time series (model I) and the mean seasonal cycle (model II)

	Model I				Model II			
	Estimate	SD	*t*	*p*	Estimate	SD	*t*	*p*
Intercept	5.3937	2.3657	2.280	< 0.05	25.9312	7.4417	3.485	< 0.05
T(0, 0)	− 0.1432	0.0439	− 3.259	< 0.01	− 0.2167	0.0859	− 2.523	< 0.05
T(6, 4)	− 0.2173	0.0398	− 5.453	< 0.001				
rH(1, 0)	− 0.0686	0.0235	− 2.915	< 0.01	− 0.3110	0.0846	− 3.676	< 0.05
Hare	0.0076	0.0017	4.325	< 0.001				
Factor(season) II	2.6083	1.1723	2.225	< 0.05	3.0718	1.4277	2.152	< 0.1
Factor(season) III	2.5290	1.2095	2.091	< 0.05	3.3341	1.4626	2.280	< 0.1
Factor(season) VI	3.5022	1.1741	2.983	< 0.01	3.7859	1.4449	2.620	< 0.05

For each explanatory variable, the parameter estimate, the standard error SE, the *t* value (test statistics), and the *p* value (significance) are given. Note that factor(season) I is not listed, as it is defined as default. Parameters T(6, 4) and Hare determining the year-to-year variation of the tick density are not needed in model II (mean seasonal cycle)

**Fig. 4:**
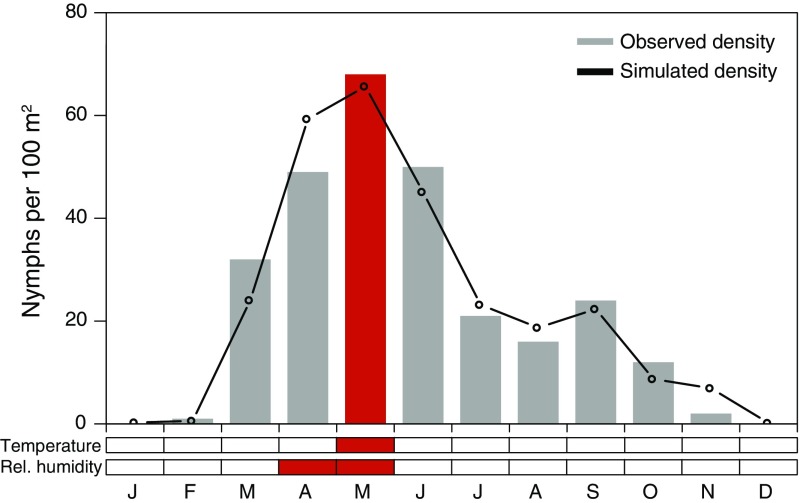
Mean monthly nymphal tick density in Haselmühl (Germany). Observations are shown as grey bars and simulations as lines. To illustrate the climatic variables determining the density in May, the mean temperature in May as well as the mean relative humidity from April to May are highlighted in red

A second model was developed to explain the mean monthly density of nymphal ticks. In order to assess the question which variables define the seasonal cycle, the procedure described above was repeated. The mean monthly density was described using only the month-to-month temperature T(0, 0), the averaged relative humidity of the month of the flagging event and 1 month previously rH(1, 0), and the four seasons as categorical variables (Table 1, Fig. 4). A marked higher ${R}^2$ of 95.6% and a goodness-of-fit of RMSE of 5 ticks per month were calculated. Compared to the mean peak tick density of about 70 ticks per 100 m$^2$ this error is extremely low.

## Discussion

A time series of monthly questing nymphal *I. ricinus* flagged in Haselmühl (Germany) over a period of eight consecutive years was presented. The length of this time series is unique for Germany. Comparable or longer time series have been published for e.g. *I. scapularis* with 8 (Schulze et al. 2009), 14 (Berger et al. 2014) or 25 years (Hayes et al. 2015), but with markedly lower flagging intervals and occasional gaps between sampling years. However, for quantitative statements, e.g. regarding the impact of climate change on tick densities, longer periods are needed. Basically, several methods are available for monitoring tick activity: flagging/dragging (Estrada-Peña et al. 2013), collecting from small mammals (Pfäffle et al. 2011), or monitoring on field plots (Dautel et al. 2008). All methods and their variations have advantages and disadvantages as discussed by Dobson (2013) or Mays et al. (2016). Nevertheless, the flagging method gives the best insights into long-term tick population dynamics and activity. Although flagging is the most common method, it is always a snapshot of the current situation influenced by environmental variables (Dantas-Torres et al. 2013). It will, therefore, be a challenge for the future to compile time series of up to 30 or more consecutive years of standardized tick sampling to improve our understanding of the influence of climate and environmental change on tick densities.

Here, cross correlation maps (CCMs) were introduced to identify time-lagged and interval-averaged associations between ticks and environmental variables. CCMs, primarily used for insects with multiple-generations per year, were applied for the first time to tick densities. For both the inter-annual and the seasonal cycle, temperature and relative humidity with different time lags were revealed as the determining environmental variables for nymphal tick densities. The diapause (survival, inactivity) and questing (activity, ability to find host, survival) are principally affected by both variables. Contrary, the development (rate and duration, emergence) is mainly influenced by temperature (Ostfeld and Brunner 2015). Here, the mean temperature in the month of the flagging event and the mean relative humidity of the actual month and the previous one are particularly important for both temporal scales. In addition, for the inter-annual tick density of the complete time series (model I), the temperature mean of 4–6 months prior to flagging is required for an optimal model fit. The environmental variables such as the temperature in May, the temperature mean from November to January (3 months) and the relative humidity mean from April to May (2 months) prior to flagging determine the first activity maximum in May (as marked in red in Fig. 3).

The seasonal and inter-annual variability of the nymphal tick density was also found to be affected by the abundance of hosts. Cayol et al. (2017) demonstrated that the abundance of rodent species is positively correlated with those of *I. ricinus* larvae and nymphs. For Germany, several time series were compiled e.g. for field voles (*Microtus agrestis*) or bank voles (*Myodes glareolus*) some of which go back to 1952 (Imholt et al. 2017). However, these vole data were not used in the current study, as the observation periods available for the voles did not match those of the ticks. Instead of voles, time series of the European hare (*Lepus europaeus*) were compiled from hunting statistics and shown to improve the model fit. The hunted hares were determined to be positively, but marginally, correlated with the nymphal tick densities in the following year. Although the role of hares in the transmission of pathogens, e.g. TBE virus (Palo 2014), is unclear, they are assumed to act as a reservoir or as transport hosts for ticks (Tälleklint and Jaenson 1993). The hunting statistics can be interpreted as in place of not yet determined factors such as small rodent density. Alternatively, rodent density may be estimated from beech mast data of the previous year. As demonstrated by Clement et al. (2009) and Reil et al. (2015), a year with abundant fructification is followed by a year with high rodent density, the main blood hosts for larvae (Matuschka et al. 1991). However, this approach revealed no significant improvement of both models.

Overall, this study demonstrated that using CCMs to identify time-lagged correlations enables quantitative seasonal predictions of nymphal tick densities to be made. The time lags and averaging intervals presented here should be generally applicable, at least in a crude approximation. This conclusion is supported by a first application of CCMs (not shown) using the *I. ricinus* time series collected in Prague (Czech Republic) by Daniel et al. (2015). The parameters for the two predictive models, however, must be appropriate for each new location. Furthermore, CCMs may be an elegant method to find optimal relationships between tick activity and the occurrence of human TBE cases. Without using CCMs, Daniel et al. (2010) found maximal correlations between TBE cases and the *I. ricinus* abundance 6 weeks ago, and a 1-month time lag was empirically estimated by Randolph et al. (2008). Such predictions are essential for epidemiological considerations: not only for individuals at risk of acquiring a pathogen capable of causing TBE or LB, but also for (veterinary) public health authorities.
